# Coronavirus: COVID-19 Transmission in Pacific Small Island Developing States

**DOI:** 10.3390/ijerph17155409

**Published:** 2020-07-28

**Authors:** Walter Leal Filho, Johannes M. Lütz, David N. Sattler, Patrick D. Nunn

**Affiliations:** 1 European School of Sustainability Science and Research, Hamburg University of Applied Sciences, Ulmenliet 20, D-21033 Hamburg, Germany; walter.leal2@haw-hamburg.de; 2 Department of Natural Sciences, Manchester Metropolitan University, Chester Street, Manchester M1 5GD U, UK; 3 School of Social Sciences, University of New South Wales (UNSW), Sydney NSW 2052, Australia; jluetz@chc.edu.au; 4 School of Social Sciences, CHC Higher Education, Carindale, Brisbane QLD 4152, Australia; 5 Department of Psychology, Center for Cross-Cultural Research, Western Washington University, Bellingham, WA 98225-9172, USA; 6 School of Social Sciences, University of the Sunshine Coast, Maroochydore QLD 4558, Australia; pnunn@usc.edu.au

**Keywords:** coronavirus, COVID-19, pandemic, coronavirus pandemic, economic impact, Pacific islands, Pacific region, Pacific Small Island Development States

## Abstract

Background: Pacific Small Island Developing States (SIDS) have health care systems with a limited capacity to deal with pandemics, making them especially vulnerable to the economic and social impacts of the coronavirus (COVID-19). This paper examines the introduction, transmission, and incidence of COVID-19 into Pacific SIDS. Methods: Calculate the rate of transmission (the average number of new cases per day between the first recorded case and the most recent day) and connectivity (daily direct flights to the leading airport in each selected island group) using flight history and COVID-19 transmission data. Results: Correlational analyses show that connectivity is positively related with (a) first-case dates and (b) spread rate in Pacific SIDS. Conclusion: Connectivity plays a central role in the spread of COVID-19 in Pacific SIDS. The continued entry of people was a significant factor for spread within countries. Efforts to prevent transmission by closing borders reduced transmission but also created significant economic hardship because many Pacific SIDS rely heavily on tourism and international exchange. The findings highlight the importance of exploring the possibility that the COVID-19 spread rate may be higher than official figures indicate, and present pathways to mitigate socio-economic impacts. The practical implications of the findings reveal the vulnerability of Pacific SIDS to pandemics and the key role of connectivity in the spread of COVID-19 in the Pacific region.

## 1. Introduction

The World Health Organization (WHO) declared the coronavirus (COVID-19) a pandemic on March 11, 2020. The next day, French Polynesia confirmed the first report of COVID-19 in the Pacific islands [1,2], and authorities in Pacific Small Island Developing States (SIDS) soon feared the spread of the virus. Health care systems unprepared to respond efficiently to a pandemic, inadequate water and sanitation treatment, and an influx of visitors from countries where the virus was active provided fertile ground for the spread of the virus in Pacific SIDS. Pacific SIDS countries include Fiji, Kiribati, Federated States of Micronesia, Nauru, Republic of Marshall Islands, Papua New Guinea, Samoa, Solomon Islands, Tonga, and Vanuatu. They share similar challenges, including limited resources, dependence on international trade, remote locations, fragile ecosystems, and susceptibility to natural disasters. French Polynesia was among the first Pacific SIDS to report COVID-19 in mid-March 2020, followed by Guam, Fiji, New Caledonia, Papua New Guinea (PNG) and Commonwealth of the Northern Mariana Islands (CNMI) [3,4]. By early April 2020, the WHO reported 192 confirmed cases and five deaths. Importantly, locations that serve as a hub for travelers continuing on to Pacific SIDS, such as Guam, were soon reporting COVID-19 [5]. In recognition of the serious threat, Honourable Kausea Natano, Chair of the Pacific Islands Forum and Prime Minister of Tuvalu, stated that COVID-19 “poses a real and extreme danger to the health and security of the Pacific peoples. Never before has the full Forum membership simultaneously been in crisis” [6].

To address the limited capacity of health care in Pacific SIDS, the WHO developed a six-month Pacific Action COVID-19 Preparedness and Response plan to reduce the virus spread and treat infected patients. The plan includes response activities such as screening passengers at major checkpoints, requiring potentially exposed persons to undergo at least 14 days of quarantine, and closing entry to non-residents. It also calls for the deployment of medical resources from better-equipped Pacific Island countries and territories, such Fiji and Guam, to less well-equipped ones [7].

Few studies have examined virus spread in Pacific SIDS during a worldwide pandemic. To understand how COVID-19 reached and spread within Pacific SIDS and other Pacific Island nations, the authors examined connectivity data for five island groups with recorded cases, dates for first reported case, and the rate of in-country transmission. Knowledge concerning virus spread throughout Pacific SIDS can assist government agencies and health organizations identify ways to mitigate spread.

## 2. Methods

To assess the connectivity of Pacific SIDS and other Pacific Island nations with locations that serve as a hub for international travelers, the authors determined the usual number of daily direct flights from Asia, the Americas, and Australasia for the leading hub airport. FlightRadar24 data was collected for flights on 8 April 2020 to determine flights per week, representing the longer-term situation, and for 9 September 2020, which, at the time, represented return to normal scheduling, from Expedia. The data are presented as an average number of flights per day from the region of flight origination. Virus transmission was calculated by determining the average number of new cases per day between the first recorded case and the most recent day for which data were available. The data were drawn from Johns Hopkins University Center for Systems Science and Engineering GitHub repository and online interactive dashboard [8,9] and the WHO COVID-19 tracker [10], which offer reliable and authoritative data on global COVID-19 case information [11].

## 3. Results

Table 1 presents connectivity to main hubs, the earliest reported COVID-19 case, and spread rate information (number of cases per day). The raw data in Table 1 are the average numbers of non-stop flights per week averaged from (1) the number of daily flights on 8 April 2020 (taken as pre-COVID), (2) the average number of flights per week (taken as pre-COVID), and (3) the number of daily flights projected on 9 September 2020 (which at the time was taken as return to normal scheduling) from sources presented in the Methods.

**Figure 1:**
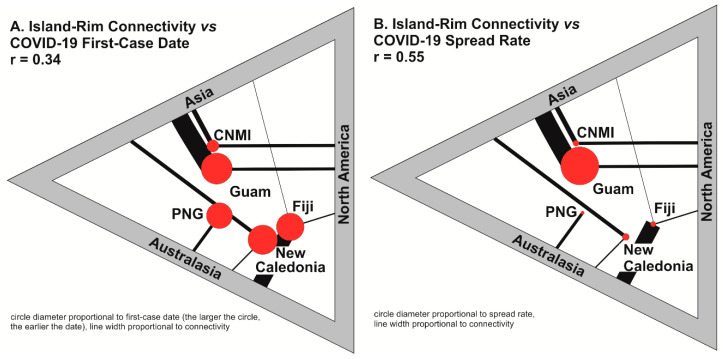
Island-rim connectivity vs. COVID-19 first-case date: (**A**) the correlation between first-case dates and connectivity; (**B**) the correlation between connectivity and spread rate.

Correlational analyses showed that there is a modest positive relationship between first-case dates and connectivity (r = 0.34, Figure 1A), suggesting that air traffic played a central role in introducing the virus to Pacific islands. This is illustrated by the contrast between well-connected Guam and poorly connected CNMI, where the virus arrived far later. The findings also suggest that Asia and Australasia sources may have been more influential than the Americas in the initial spread.

Importantly, an additional correlational analysis shows that connectivity is positively associated with spread rate (r = 0.55; Figure 1B), suggesting the continued entry of people was a significant factor for spread within country. For example, Guam has the fastest spread, where arrivals from Asia continued, comparatively, long after the earliest case; in contrast, lower levels of connectivity in CNMI and PNG help explain the slower spread rates. Asia and Australasia appear to be significant sources, but this may be a function of the imbalance of connectivity sources in the selected island groups.

Remoteness, isolation, and inaccessibility have thus far allowed several island states to escape infection. As of 2 May 2020, Kiribati, Marshall Islands, Micronesia, Nauru, Palau, Samoa, Solomon Islands, Tonga, Tuvalu, and Vanuatu have not reported any COVID-19 cases. Table 2 shows that of the top-ten least-visited countries, six are Pacific Island states. The important inference is that, in a COVID-19 world, lower numbers of international arrivals appear to convert to a lower incidence of infection.

## 4. Discussion

The connectivity-spread rate correlation (Figure 1) has important ramifications for remote SIDS. On the upside, reduced connectivity to and from islands in the Pacific implies that the arrival of both imported goods and COVID-19 may be limited or delayed. On the flip side, this may also mean that outside help, possible escape, or medical evacuations to areas with more suitable medical facilities may be more difficult. Many islands are densely populated and have inadequate sanitation and unreliable sea transport, providing fertile contextual conditions for both the spread of infection and its entrapment [12]. This underscores the crucial strategy of fending off COVID-19 in the Pacific islands in the first place.

Countries that closed their borders to tourists and other travelers and required returning citizens to self-quarantine achieved some level of success in reducing or preventing transmission. Several Pacific SIDS, including Kiribati, Nauru, Tonga, and Tuvalu, have no reported COVID-19 cases to date [13]. Other actions promoted by health officials, including recommending or requiring wearing face masks to prevent the transmission of droplets and particles from mouth and nose, and frequent hand washing, helped reduce the spread [14]. As countries reopen their borders, the current findings suggest that additional health screening measures to identify people with COVID-19 exposure who do and do not show symptoms are warranted. These assessments could occur shortly before starting travel and/or upon arrival to the destination country. Individuals with COVID-19 exposure within the past 14 days might be denied travel, required to take additional measures to prevent transmission during travel, and/or required to self-quarantine upon arrival.

### Reducing Connectivity: Socio-Economic Implications

The connectivity findings and actions to reduce the spread of COVID-19 have socio-economic implications for Pacific SIDS. As connectivity to Pacific SIDS is essential to economic development, nations dependent on connectivity (e.g., tourism, imports, and financial grants) are at particular risk. For example, in Fiji, tourism accounts for nearly 40% of its gross domestic product (GDP) and approximately 37% (direct and indirect) of all employment [15]. Efforts to prevent the transmission of the virus and promote physical distancing has resulted in a virtual shutdown of all tourism in Pacific SIDS. For example, in early April, Fiji Airways reduced 99% of its international flights (with cancellations extended into summer), followed by suspension of domestic flights a week later; other carriers also suspended travel to Pacific Island nations. International tourist cruise ships have been prohibited indefinitely from docking in Fiji and other Pacific SIDS. GDP is predicted to decrease by at least 4.9%, and unemployment throughout the region is rising as a consequence of tourism stoppage. An economic recession is likely in Pacific SIDS [16].

The ramifications are rippling through sectors that support the tourism industry, including agriculture, transportation, retail, lodging, food, and recreation. The loss of tourism in Vanuatu, for example, is threatening to reverse 15 years of steady growth. Countries less reliant on tourism may be more resilient, such as Kiribati, Nauru, and Tuvalu. Papua New Guinea is more dependent on mining and petroleum production than tourism. The economies of some Pacific SIDS rely on public trust funds (e.g., Kiribati and Nauru) but these may be at risk if economic-fiscal instability expands [16]. As travel bans extend into the summer months, there is uncertainty regarding the final economic toll. Inflation is expected to increase as the demand for goods and services decline. Poverty in Pacific and East Asian countries is likely to increase by approximately 11 million in 2020, and about 24 million people currently living in poverty will find it more difficult to move beyond it [17]. 

To make matters worse, from 6 April 2020 to 10 April 2020, Cyclone Harold created damage in several Pacific Island nations. It made landfall in Vanuatu as a Category 5 storm, slightly decreased in strength and brushed Fiji, and then increased in strength and struck Tonga during a king tide. These nations reported extensive losses to businesses (including tourist resorts), homes, crops, and communication capacity. Cyclone Harold came while Vanuatu, Fiji, and Tonga were recovering from recent catastrophic storms: Cyclones Pam (2015), Winston (2016), and Gita (2018) [18]. Cyclone Harold exacerbated immediate economic hardships brought on by COVID-19 and created new challenges to long-term recovery due to both the virus and the cyclone. Climate change is responsible, in part, for the increased strength of cyclones in recent years by increasing ocean temperatures, which provide fuel for cyclones [19].

Fishing contributes to Pacific Island economies, and the industry may decline due to restricted connectivity and COVID-19 supply chain disruptions, restrictions on international sales of fresh fish, and boat crew health issues related to the virus. Illicit fishing and the transportation of unlawful cargo by smugglers and poachers may increase as critical personnel are reassigned from policing fisheries to addressing national COVID-19 issues, thereby reducing the likelihood of seizure [20].

Economic aid and assistance provided by other nations can help mitigate adverse economic consequences in Pacific SIDS. Increased international cooperation and public–private partnerships are vital to producing and supplying necessary medical supplies and promoting stability, as are open trade policies [17]. To address potential economic instability and advance the cooperation, assistance, and distribution of medical and humanitarian aid, on 7 April 2020, the Pacific Island Forum member nations established the Pacific Humanitarian Pathway on COVID-19, and underscored the importance of “te fale-pili,” a Tuvaluan belief common throughout Pacific Island cultures that neighbors have a moral responsibility to safeguard and help one another [5]. Understanding the relationship of connectivity with the spread and the importance of taking appropriate preventive measures is essential to reduce or eliminate COVID-19 spread.

The study has a few limitations. Because Pacific SIDS and nations around the world had a limited supply of testing kits for COVID-19, the full number of cases may be underreported in the data. The study is correlational, and the full scope of spread may be underrepresented. Nonetheless, the evidence gathered in this paper may support future policies to mitigate the spread of pandemics in the Pacific region.

## 5. Conclusions

The connectivity-spread rate analysis presented in this paper offers pathways to delay or mitigate the arrival of COVID-19 in Pacific SIDS by reducing connectivity to/from and between/within islands. Measures to reduce connectivity should consider allowing for the appropriate introduction of outside help, reunification of citizens (with appropriate quarantine upon return), and medical evacuations to areas with more suitable medical facilities. Decisions to reduce connectivity should carefully consider ways to mitigate socio-economic harm.

The findings also highlight the importance of exploring the possibility that the COVID-19 spread rate may be higher than official figures indicate. Indeed, “highly likely gross under-detection and underreporting of mild or asymptomatic cases inevitably throws severe disease courses calculations and death rates out of context, distorting epidemiologic reality” [21].

While the apparent absence of COVID-19 from a portion of the Pacific Islands region until now gives cautious hope, we should remember that “absence of evidence about a problem does not imply evidence of absence of a problem” [22]. For example, in the United States, community transmission remained undetected until infection rates were confirmed through ramped-up testing regimes. As to the future, it is hoped that the measures in place across most Pacific Island countries, such as physical isolation and the partial shutdown, may avert a potential health and economic disaster, providing a basis for future recovery.

The practical implications of the findings are twofold. Firstly, they reveal an association between connectivity and the spread of COVID-19 in the Pacific region, and that less connected regions were among the least affected. Secondly, they outline the vulnerability of Pacific nations to pandemics. Such vulnerability results not only from the fragility of health systems on Pacific SIDS that are not well equipped to handle high numbers of patients, but also due to the economic problems as a result of measures to reduce virus transmission, including the closure of country borders and stay-at-home or lockdown mandates. These efforts place the economic systems of Pacific SIDS under substantial distress and can lead to hardships and a worsening of social problems.

Moving forward, there are some pathways which Pacific nations may follow in order to better cope with the prospects of a widespread COVID-19 pandemic. This entails measures aimed at:

- Addressing how to mitigate social and economic impacts of the outbreak response related to health systems;
- Identifying non-intended consequences of epidemic control decisions;
- Providing answers to social dynamics of the outbreak and the related public health response;
- Identifying the means to address inequalities that may be exacerbated by the epidemic, such as limited access to medical services and treatments, especially among the elderly.

A pathway to reduce the vulnerability of the region to COVID-19 should also analyze the effects and efficiency of these responses (including resilience factors), democratic governance, multi-level cooperation, the critical gaps and the various exit strategies, with their underlying methodologies and the prospects for regional adaptations. It will also be helpful if Pacific countries develop guidelines and best ‘next practices’ and implement interventions to mitigate impacts and boost well-being.

Finally, research on virus outbreak responses across the region and the impacts on human behavior and social dynamics is needed to help guide future responses. These efforts should take into account societal and cultural structures, health system preparedness and resilience, population densities, population risk groups, climate, and pollution.

## Figures and Tables

**Table 1:** Pacific Island group connectivity, the earliest reported COVID-19 case, and the spread rate.

Airport (Pacific Island Group)	Flights from Asia	Flights from Americas	Flights from Australasia	Earliest COVID-19 Case Date	Spread Rate (Cases Per Day)
Guam (USA)	17.43	3.00	0.00	16 March 20	5.50
Saipan (CNMI)	4.71	3.14	0.00	31 March 20	0.86
Port Moresby (PNG)	0.00	0.00	3.00	20 March 20	0.06
Noumea (New Caledonia)	3.71	0.00	1.29	18 March 20	1.00
Nadi (Fiji)	0.14	1.29	14.86	19 March 20	0.79

Note: Movements between Pacific Islands are not of interest in this research and have only a negligible impact on spread within the overall snapshot shown in Table 1 and Figure 1.

**Table 2:** World’s top-ten least-visited countries (fewest international arrivals according to the UN World Tourism Organization; 2018—the most recent year available) and COVID-19 infection rates.

Country	Arrivals (2018)	COVID-19 Infections (as of 11 April 2020)
Fewest arrivals		
Comoros	35,000	0
Sao Tome and Principe	33,400	4
Mauritania	30,000	7
Solomon Islands *	27,900	0
American Samoa *	20,200	0
Micronesia Fed. Sts. *	19,200	0
Mali	14,000	87
Kiribati *	7100	0
Marshall Islands *	6800	0
Tuvalu *	2700	0

* = Pacific Island state. Table based on data excerpted from World Bank (2019) and Johns Hopkins University. It should be noted that the data refer to figures gathered by the time the paper has been prepared and offers a profile of the trends seen then. The number of cases has increased since. The important upshot is that in a COVID-19 world, lower numbers of international arrivals appear to convert to a lower incidence of infection. Clearly, characteristics such as remoteness, isolation, and inaccessibility bode well for ‘social distancing’ outcomes in the Pacific Island region, given that little-visited islands may be seen as natural ‘self-isolators’.
